# Incidence of Stress-Induced Hyperglycemia in Acute Ischemic Stroke: A Systematic Review and Meta-Analysis

**DOI:** 10.3390/brainsci13040556

**Published:** 2023-03-26

**Authors:** Haofuzi Zhang, Kangyi Yue, Zijian Jiang, Xiuquan Wu, Xin Li, Peng Luo, Xiaofan Jiang

**Affiliations:** 1 Department of Neurosurgery, Xijing Hospital, Fourth Military Medical University, Xi’an 710032, Chinapengluo@fmmu.edu.cn (P.L.); 2 Institute of Neurosurgery of People’s Liberation Army of China (PLA), PLA’s Key Laboratory of Critical Care Medicine, Xijing Hospital, Fourth Military Medical University, Xi’an 710032, China; 3 Department of Hepato-Biliary Surgery, Xijing Hospital, Fourth Military Medical University, Xi’an 710032, China; 4 Department of Anesthesiology, Xijing Hospital, Fourth Military Medical University, Xi’an 710032, China

**Keywords:** stress-induced hyperglycemia, acute ischemic stroke, incidence, systematic review, meta-analysis

## Abstract

The aim of this study was to systematically evaluate the incidence of stress-induced hyperglycemia (SIH) in acute ischemic stroke (AIS). Studies that reported SIH incidence in AIS and examined risk factors for SIH and non-SIH patients were systematically searched in PubMed, Embase, Cochrane Library, and Web of Science from the inception of each database to December 2021. Article screening and data extraction were performed by two independent reviewers according to the inclusion and exclusion criteria. The quality of the included studies was assessed using the Newcastle–Ottawa Scale (NOS), and meta-analysis was performed using Stata. A total of 13 studies involving 4552 patients (977 in the SIH group and 3575 in the non-SIH group) were included. Meta-analysis showed that the incidence of SIH was 24% (95% CI: 21–27%) in the total population, 33% (14–52%) in North America, 25% (20–29%) in Europe, and 21% (12–29%) in Asia. Subgroup analysis by year of publication revealed that the pooled incidence of SIH was 27% (22–32%) in studies published before 2010 and 19% (14–24%) in those published after 2010. SIH is relatively common in AIS and poses a serious public health problem. Therefore, more emphasis should be placed on the prevention and control of SIH in AIS.

## 1. Introduction

Cerebrovascular disorders are frequently confronted by physicians. Stroke, the manifestation of cerebrovascular disorders that most commonly happen, is the leading cause of hospitalization for neurologic disease as well as disability and death in adults worldwide, which is characterized by acute onset, rapid progression, and poor prognosis [1]. Stroke is divided into hemorrhagic or ischemic. Ischemic stroke is caused by the occlusion of vessels that reduces the blood supply to the brain. Hemorrhagic stroke occurs because of cerebral or subarachnoid hemorrhage after the cerebral vascular rupture [2,3]. In ischemic stroke, the insufficient blood supply to the brain tissue firstly results in reversible loss of tissue function and, given enough time, infarction with loss of neurons and supporting structures [4]. Acute ischemic stroke (AIS) accounts for 60–80% of all stroke cases, and stress-induced hyperglycemia (SIH) is a common complication of AIS [5]. The causes of poor pre-treatment in AIS patients with SIH include elevated blood glucose levels, increased blood flow resistance, and reduced brain metabolic rate. In addition, hyperglycemia after cerebral ischemia and hypoxia increases anaerobic metabolism in the brain and accumulation of lactic acid, leading to brain edema and brain injury. The combination of hypoxia, and acid poisoning, causes swelling and disintegration of mitochondria, which leads to a vicious cycle of energy metabolism and, thereby, irreversible neurological damage [6]. Admission hyperglycemia was reported to be associated with poor prognosis and high 3-month mortality in AIS patients as well as patients receiving thrombolytic therapy. Current studies have not confirmed the relationship between hyperglycemia and long-term AIS prognosis, but admission hyperglycemia is associated with higher mortality within 5 years after AIS [7]. Non-diabetic patients admitted to the hospital after AIS with blood glucose levels between 6.1 and 7 mmol/L had a 3.8-fold increase in the risk of short-term death. However, both hyperglycemia and hypoglycemia can lead to poor prognosis in diabetic patients with AIS, and the prognosis is better in patients with an initial blood glucose of 3.7–7.3 mmol/L [8].

Previous studies showed that SIH may be associated with the clinical prognosis of stroke and is affected by basal glucose levels due to diabetes management and diet [9]. SIH occurs when the blood glucose level is increased under stress. Some scholars believe that a post-admission fasting glucose of ≥6.9 mmol/L or random blood glucose of ≥11.1 mmol/L can be used as the criterion for diagnosing SIH. However, according to clinical trials of intensive insulin therapy, Stress hyperglycemia is diagnosed at 1 mmol/L [6,10]. The pathogenesis of SIH is relatively complex. Clinically, it is believed that SIH is caused by stress-induced massive cytokine release and neuroendocrine disorders. Stress-induced glucose metabolism is primarily regulated via the hypothalamic–pituitary–adrenal axis, immune–neuroendocrine axis, and hypothalamic–pituitary–thyroid axis, which are all related to catabolism and stress [11]. The main mechanisms involved are as follows [12,13]: (1) the increase in growth hormone secretion in the body after the body produces following a stress response, switch of, the release and secretion of growth hormone will be increased to a certain extent, and the energy source will be transferred from sugar metabolism to fat metabolism, and the inhibition of glucose utilization and uptake of glucose by peripheral tissues will be inhibited, thus promoting the progression of SIH; (2) catecholamine hormones consist mainly of norepinephrine and epinephrine; (3) increased secretion of corticosteroids, an important reaction in stress response, is increased secretion of glucocorticoids; (4) the body’s immune cells and various cytokines also play important roles in the stress response, such as interleukin (IL)-1, leptin, interleukinIL-6, and tumor necrosis factor (TNF)-α. The patients with higher blood sugar glucose values and patients under stress have more serious conditions, worse prognosis of patients, and higher mortality. Although transient SIH can occur in patients, its persistence will affect tissue repair, lead to immune suppression of immune function, induce body infection, amplify systemic inflammatory response, delay brain function recovery, etc., and increase the mortality and incidence of multiple organ dysfunction. Therefore, clinical attention should be paid to the existing presence of SIH [14,15]. Transient stress reaction is closely related to hyperglycemia patients’ disease, nutritional supply, multiple organ dysfunction, and various complications. Stress hyperglycemia has potential harm to the body, such as disturbance of water, electrolyte, and acid–base balance, damage to liver tissue, myocardium, and brain tissue, and an increase in infection. The higher the blood sugar value of patients under stress, the more serious the disease, the worse the prognosis of patients, and the higher the mortality rate. Although transient stress hyperglycemia occurs in patients, its persistence will affect tissue repair, lead to suppression of immune function, induce body infection, amplify systemic inflammatory response, delay brain function recovery, etc., and increase the mortality and incidence of multiple organ dysfunction. Therefore, clinical attention should be paid to the existence of stress hyperglycemia. A study of 2030 hospitalized patients showed that the blood glucose level of patients can significantly affect the prognosis of patients to a certain extent, and the mortality rate of newly diagnosed hyperglycemia patients was significantly higher than that of patients with a history of diabetes.

In addition, a hyperglycemic environment has been reported to aggravate cerebral tissue damage and edema, increase the area of infarction [16], and decrease the efficacy of thrombolysis and thrombectomy, which consequently affects brain function recovery and increase the disability and mortality rates of inpatients [17,18]. A retrospective study by Whiteley et al. [19] showed that hyperglycemia was related to hemorrhagic transformation in patients with acute stroke after thrombolysis, and the relative risk of intracranial hemorrhage after thrombolysis is increased by 1.10% for every 1 mmol increase in blood glucose. Epidemiological studies have shown that a history of long-term hyperglycemia can increase infarct size, promote the risk of reperfusion, and thus aggravate stroke lesions and even increase mortality. Hyperglycemia is harmful to progressive stroke [20]. It has been reported that SIH is a strong predictor for poor prognosis and high mortality after thrombectomy. However, the incidence of SIH in AIS significantly varies among studies due to differences in definition, study subjects, questionnaire design, and diagnostic criteria [21]. Therefore, we conducted a systematic review and meta-analysis to evaluate the incidence of SIH in AIS in order to provide insight into the development of health equity strategies for special populations.

## 2. Materials and Methods

The review was guided by the recommendations from the Preferred Reporting Items for Systematic Reviews and Meta-Analyses (PRISMA), the specific guidelines for reporting meta-analyses of observational studies, and guidelines for undertaking systematic reviews of incidence and prevalence studies.

### 2.1. Inclusion and Exclusion Criteria

Only articles published in English were included. Titles and abstracts were screened for initial study inclusion. The full texts of potential articles were thoroughly examined when the title and abstract were insufficient to determine whether the study met the inclusion criteria. In instances where there was more than one publication resulting from the same clinical trial, we selected the most complete publication for analysis.

#### 2.1.1. Study Type

Original epidemiological cohort studies written in English were included in the meta-analysis.

#### 2.1.2. Subjects

Patients who met the clinical diagnostic criteria for AIS accompanied with or without SIH were included. Patients with a history of diabetes or glucose-lowering treatment were excluded.

#### 2.1.3. Exposure

The exposure group consisted of AIS patients with SIH, and the control group consisted of AIS patients without SIH.

#### 2.1.4. Outcome

Outcomes of interest were the incidence of SIH in AIS.

#### 2.1.5. Exclusion Criteria

The exclusion criteria were (1) duplicate records; (2) articles with unextractable data; (3) sample size < 10; (4) exact time and location of the study were not reported.

### 2.2. Search Strategy

The meta-analysis was performed according to the Preferred Reporting Items for Systematic Reviews and Meta-Analysis (PRISMA) guidelines (Supplementary Materials, PRISMA_2020_checklist). Relevant articles were searched in computerized literature databases (PubMed, Embase, Cochrane Library, and Web of Science) from inception to 20 July 2022, using the MeSH and entry terms “Ischemic Stroke”, “Ischemic Strokes”, “Stroke, Ischemic”, “Ischaemic Stroke”, “Ischaemic Strokes”, “Stroke, Ischaemic”, “Acute Ischemic Stroke”, “Acute Ischemic Strokes”, “Ischemic Stroke, Acute”, “Stroke, Acute Ischemic”, “Hyperglycemia”, and “Hyperglycemias”. The PubMed search using the following terms: ((“Ischemic Stroke”[Mesh]) OR ((((((((((Ischemic Stroke[Title/Abstract]) OR (Ischemic Strokes[Title/Abstract])) OR (Stroke, Ischemic[Title/Abstract])) OR (Ischaemic Stroke[Title/Abstract])) OR (Ischaemic Strokes[Title/Abstract])) OR (Stroke, Ischaemic[Title/Abstract])) OR (Acute Ischemic Stroke[Title/Abstract])) OR (Acute Ischemic Strokes[Title/Abstract])) OR (Ischemic Stroke, Acute[Title/Abstract])) OR (Stroke, Acute Ischemic[Title/Abstract]))) AND ((“Hyperglycemia”[Mesh]) OR (((Hyperglycemia[Title/Abstract])) OR (Hyperglycemias[Title/Abstract]))). The detailed search history of PubMed, Embase, Cochrane Library, and Web of Science can be found in Supplementary Materials (Search History). Related journals and references in the included articles were also manually searched.

### 2.3. Article Screening, Data Extraction, and Quality Assessment

Two independent evaluators independently screened the literature for data extraction. By reading the title and abstract of the literature, as well as the full text of the literature, literature screening was conducted directly for research studies that were easy to judge. For the literature that can be included with objections, consult the relevant teachers’ opinions and screen by directly downloading and reading the full text. During the screening process, strictly follow the inclusion and exclusion criteria, extract the observation indicators of the two groups of studies, and cross-check the extracted data to ensure consistency of the extracted data. Search results were uploaded into Endnote (Clarivate Analytics, Philadelphia, PA, USA), and duplicates were removed. Two reviewers independently screened titles and abstracts against eligibility criteria. After initial screening, two reviewers independently assessed the full text of the retrieved articles for compliance with eligibility criteria. A PRISMA flow chart of the study selection procedure was created.

Data were extracted using a customized data extraction form, including (1) general information about the study, such as first author and year of publication; (2) study design and key factors for quality assessment; (3) general characteristics of patients, such as gender, age, sample size, and incidence of SIH; (4) outcome measures and their effect size.

The quality of the case-control studies was evaluated using the Newcastle–Ottawa Scale (NOS) [22]. In case of disagreement, a consensus was reached through discussion with a third party. The NOS scale consisted of 3 dimensions and 8 items: 4 items for object selection, 1 item for intergroup comparability, and 3 items for outcome measurement. Except for the item of comparability, the maximum score is 2 points, other items can be scored 1 point, and the score range is 0~9 points. The higher the total score, the higher the quality of the study. If multiple queues are included in the literature, they are scored separately.

### 2.4. Statistical Analysis

Outcome measures were meta-analyzed using Stata 16.0. A summary table was used to display data extracted from eligible studies. Meta-analyses of cumulative incidence and prevalence were conducted. The unit of analysis for questions 1–3 was the patient, but for question 4 it was the PI. Prior to pooling data from individual studies, double arcsine transformation was used to stabilize the variance. This method has been shown to produce fewer bias results than logit transformation. The Cochran Q test (*p* < 0.05 was considered significant) and I2 (>50% reflecting heterogeneity) were used to assess the heterogeneity between studies. The random effect model was used due to heterogeneity among studies, and because it was greater than 75% for most analyses, only 95% CIs and no point estimates were reported. For ease of interpretation, results are reported after transforming the effect sizes back to natural proportions. To explore the heterogeneity among studies, sensitivity analyses were carried out. Sensitivity analyses were undertaken, including studies excluding stage, each stage of PI, studies with low risk of bias, and studies that used skin inspection to identify PI. Both data were represented by 95%CI, and *p* < 0.05 was considered statistically significant. The stability of the research results was investigated by further sensitivity analysis. Publication bias was evaluated by Egger’s linear regression. A *p* < 0.05 is considered statistically significant.

### 2.5. Subgroup Analysis

For the incidence rate, subgroup analysis was conducted according to indicators such as country, region, and year of publication to explore more possible results.

## 3. Results

### 3.1. Article Search Results and General Characteristics of Included Studies

**Figure 1:**
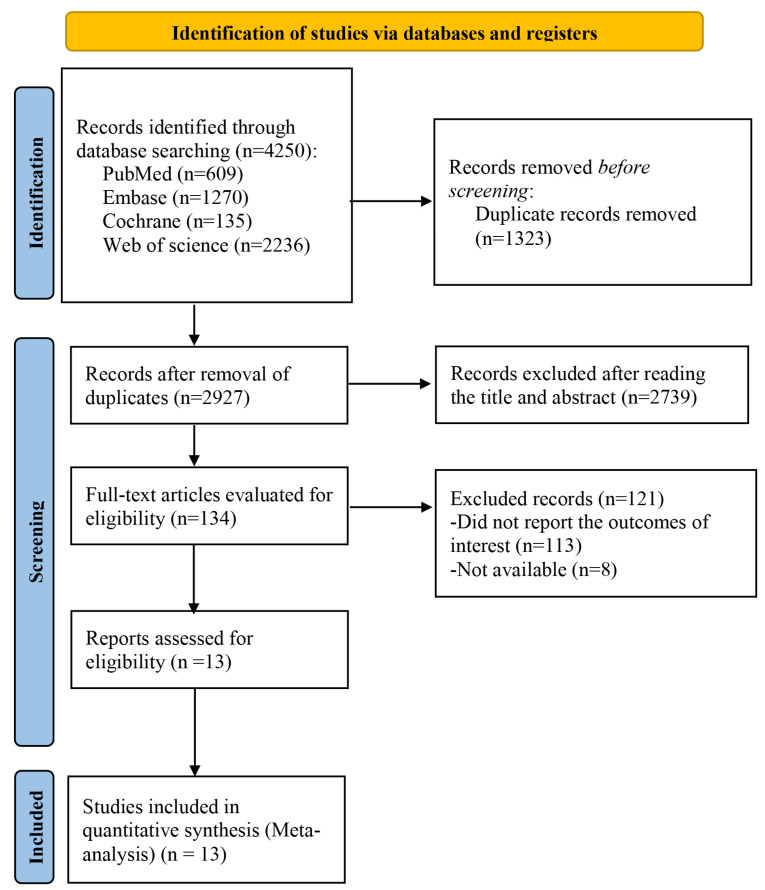
Literature Screening Flow Chart.

We initially identified 4250 English articles and removed 1323 duplicates using Endnote. Title, abstract and full-text screening was performed on the remaining 2927 articles according to the inclusion/exclusion criteria. The flow diagram of the study process is shown in Figure 1. A total of 13 retrospective cohort studies [23,24,25,26,27,28,29,30,31,32,33,34,35] were finally included. These included studies originating from Canada, Italy, Poland, Australia, India, Finland, the United States, Japan, China, Greece, and the Netherlands. Of the 4552 patients involved in these studies, the age of patients ranged from 37.1 to 87.5 years, and 41.45% were males. The NOS score was 9 for all articles, indicating high methodological quality. The general characteristics of the articles are shown in Table 1.

### 3.2. Meta-Analysis

All figures and tables should be cited in the main text as Figure 1, Table 1, etc.

#### 3.2.1. Incidence of SIH in AIS

**Figure 2:**
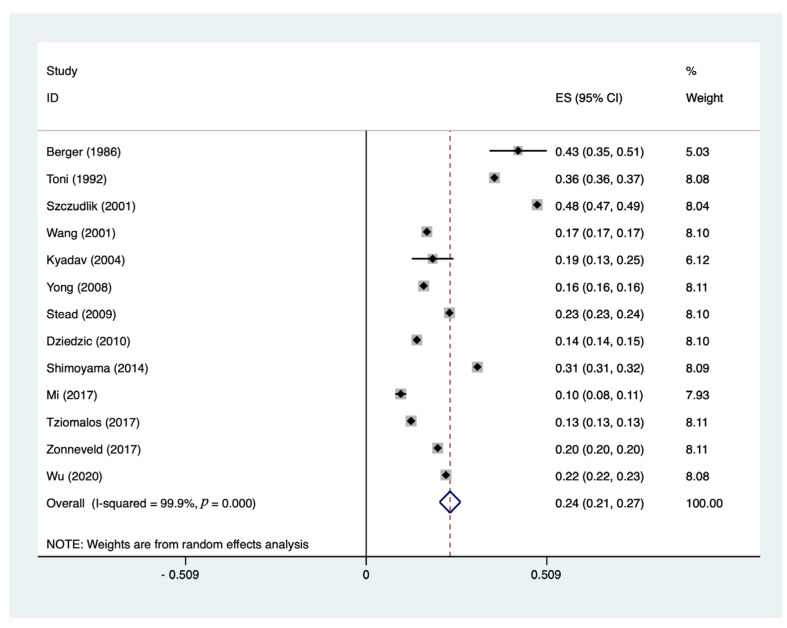
Forest plot of SIH incidence. It includes individual weighted effect size Hedge’s g represented as black diamonds, and the whiskers represent the 95% confidence intervals. The pooled weighted effect size and 95% CI are presented at the bottom with an empty diamond. Refs [23,24,25,26,27,28,29,30,31,32,33,34,35] mentioned.

**Figure 3:**
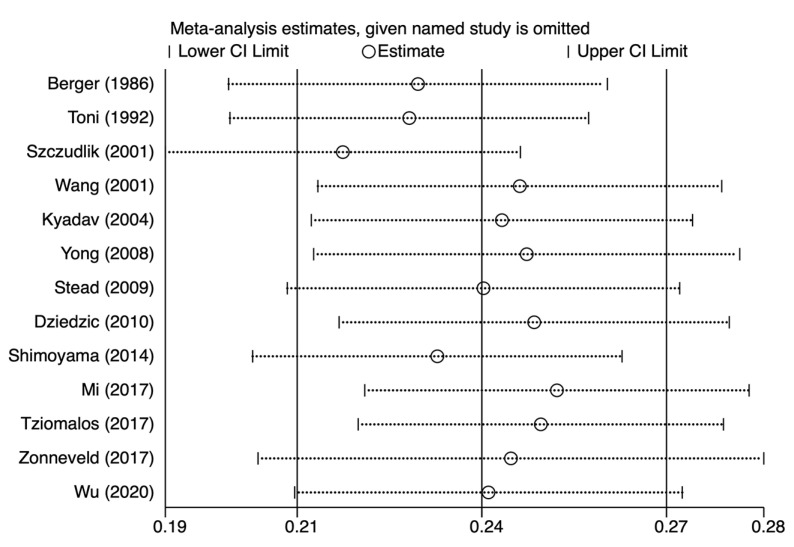
Sensitivity analysis plot. Refs [23,24,25,26,27,28,29,30,31,32,33,34,35] mentioned.

**Figure 4:**
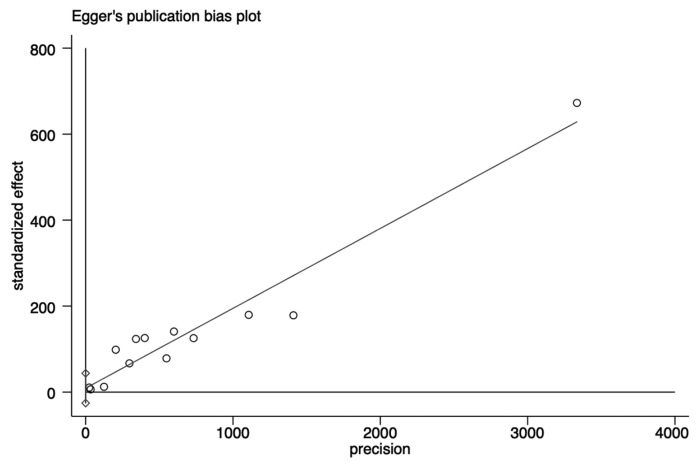
Publication bias plot. Each circle represents a retrospective cohort study [23,24,25,26,27,28,29,30,31,32,33,34,35].

Of the 4552 patients, 977 were in the SIH group, and 3575 were in the non-SIH group. Meta-analysis showed that the incidence of SIH was 9.76–48.22%, and the pooled incidence was 24% (95% CI: 21–27%). There was significant heterogeneity in SIH incidence among studies (*I*^2^ = 99.9%, *p* < 0.001), which may be attributed to differences in region and year of publication (Figure 2). Sensitivity analysis revealed that the effect size after the exclusion of each study was within the range of pooled effect size, indicating that the meta-analysis results were reliable (Figure 3). There was no publication bias among the studies (Egger’s test *p* = 0.579) (Figure 4).

#### 3.2.2. Subgroup Analysis

**Figure 5:**
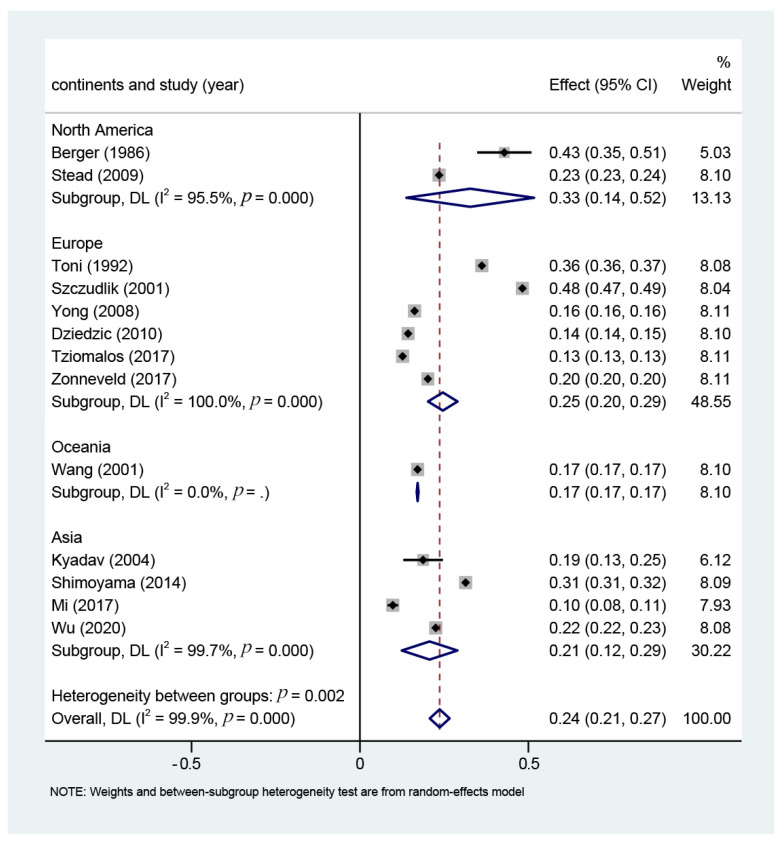
Subgroup analysis based on continent. It includes individual weighted effect size Hedge’s g represented as black diamonds, and the whiskers represent the 95% confidence intervals. The overall weighted effect size and 95% CI are presented at the bottom of each subgroup and as a whole with an empty diamond. Refs [23,24,25,26,27,28,29,30,31,32,33,34,35] mentioned.

**Figure 6:**
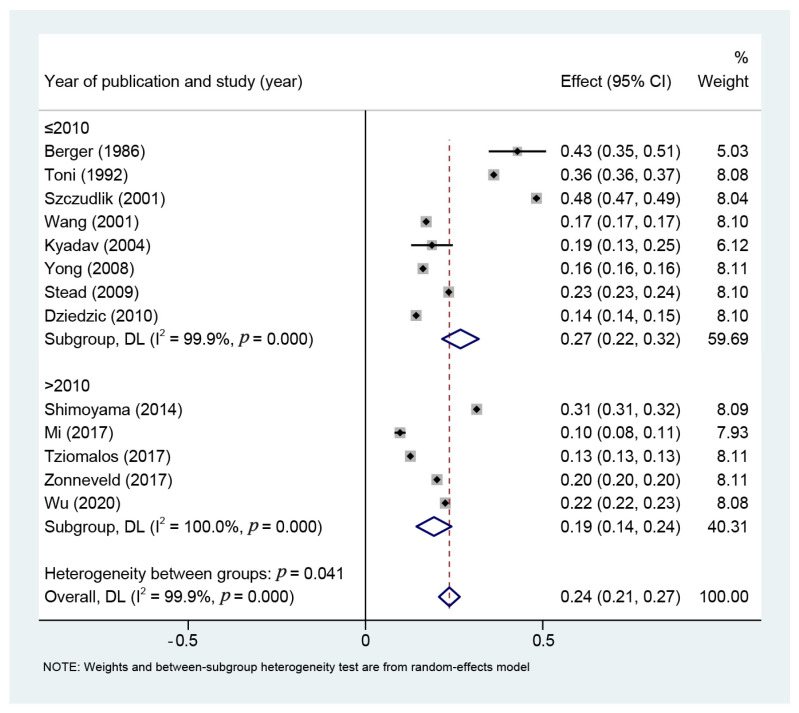
Subgroup analysis based on year of publication. It includes individual weighted effect size Hedge’s g represented as black diamonds, and the whiskers represent the 95% confidence intervals. The overall weighted effect size and 95% CI are presented at the bottom of each subgroup and as a whole with an empty diamond. Refs [23,24,25,26,27,28,29,30,31,32,33,34,35] mentioned.

Given the relatively high heterogeneity in SIH incidence, subgroup analysis according to continent and year of publication was performed. The results showed that the incidence of SIH was 33% (14–52%) in North America, 25% (20–29%) in Europe, and 21% (12–29%) in Asia (Figure 5). In addition, the pooled incidence of SIH was 27% (22–32%) in studies published prior to 2010 and 19% (14–24%) in those published after 2010 (Figure 6).

## 4. Discussion

### 4.1. Findings

Our systematic review and meta-analysis of 13 cohort studies involving 4552 patients (997 in the SIH group and 3575 in the non-SIH group) showed that the pooled incidence of SIH in AIS is 24% (21–27%), which is lower than the 40% reported in previous studies [36,37]. Despite the presence of heterogeneity among the included studies (*I*^2^ = 99.9%, *p =* 0.00), our sensitivity analysis indicated that the heterogeneity had no substantial impact on the results. In addition, the meta-analysis showed that the incidence of SIH is different among continents. Subgroup analysis revealed a higher SIH incidence in North America (33%) compared to Europe (25%) and Asia (21%), which may be attributed to diet and race. However, it is important to note that there was still a considerably high heterogeneity in SIH incidence after subgrouping [38]. On the other hand, we found that the pooled incidence of SIH was lower in studies published after 2010 (19%, 95% CI: 14–24%) than those published before 2010 (27%, 95% CI: 22–32%). This may be associated with the advancement in medical techniques, which have allowed early diagnosis and prevention of SIH in AIS, thus reducing the incidence of SIH in AIS [39]. Our results suggest that the incidence of SIH in AIS will continue to decrease in the future as medical techniques continue to advance. SIH is a common complication of AIS. It was reported that patients with hemorrhagic stroke have much higher blood glucose than those with ischemic stroke. There are several mechanisms by which hyperglycemia leads to poor prognosis after AIS. First, hyperglycemia may be directly toxic to ischemic brain tissue. Although the mechanisms are not fully understood, lactic acid accumulation and intracellular acidosis are present in ischemic brain tissue. These neurotoxic effects may be particularly important in the ischemic penumbra. Hyperglycemia promotes the development of intracellular acidosis in the ischemic penumbra, leading to a larger cerebral infarction area. Thus, hyperglycemia can promote the development of potentially viable neurons for cerebral infarction. Second, blood glucose metabolism disorder develops in different ways, such as in patients who have not been diagnosed with diabetes but present SIH, have abnormal glycopenia, or have not been diagnosed with diabetes under non-stress conditions. These patients suffered more ischemic injury after acute ischemic stroke than those without stress hyperglycemia, resulting in potentially more extensive cerebrovascular lesions. Third, endothelial dysfunction, including the lack of insulin in patients with hyperglycemia, leads to decreased peripheral glucose uptake and elevated circulating free fatty acids, which may impair endothelium-dependent vasodilation. Even hyperglycemia in non-diabetic patients is associated with endothelial dysfunction, another potential mechanism driving the cerebrovascular disease. Fourth, hyperglycemia disrupts the blood–brain barrier and promotes hemorrhagic transformation after cerebral infarction. An observational study of 138 patients with and without prior diabetic stroke treated with recombinant tissue plasminogen activator showed that higher blood glucose levels were associated with a higher risk of cerebral hemorrhagic transformation. Hyperglycemia may occur in patients with severe or fatal stroke due to the release of large amounts of stress hormones, such as catecholamine. In fact, the strength of the positive association between hyperglycemia and mortality increases as stroke severity increases. Therefore, early intervention is necessary for acute ischemic stroke patients with acute hyperglycemia.

### 4.2. Clinical Significance

Type 2 diabetes patients have at least a 2-fold increase in the risk of developing AIS, and hyperglycemia in AIS patients is associated with high disability and mortality rates [40].

The risk factors and pathogenesis of progressive stroke are complex and diverse. The increase and decrease in blood glucose levels in progressive stroke are mainly caused by (1) undiagnosed diabetes mellitus or abnormal glucose metabolism; (2) stress response: acute stroke stimulates the sympathetic nerve, increases glycotropic hormone level, reduces glucose utilization, and thereby results in elevated blood glucose, insulin resistance or insufficient insulin secretion. When general stress factors are eliminated, blood glucose level can be restored to baseline; (3) lack of insulin antagonism in high glucose water during intravenous fluid replacement for inpatients with iatrogenic acute cerebral apoplexy; (4) placeholder effect: the probability of post-stroke hyperglycemia is significantly higher in patients with stroke near the midline than in those far from the midline, with a decreasing incidence from the brainstem > thalamus > basal ganglia > lobe. The closer the stroke site is to the midline structure, the greater the stimulation of the hypothalamic–pituitary–target gland axis and the sympathetic nerve fibers that are present in the brain stem network structure. The release of glycotropic hormones that activate various neuroendocrine systems ultimately increases blood glucose levels; (5) reduced hypoglycemia-induced feedback in patients with diabetes mellitus combined with autonomic nervous dysfunction, thus increasing the risk of severe hypoglycemia; (6) hypoglycemia resulting from the inability to consume food, such as loss of appetite or coma, in diabetic patients, especially those who are taking hypoglycemic drugs, or the failure to manage diabetes in a timely manner. Studies have shown that SIH is associated with stroke severity and inflammation, which is consistent with its potential mechanism and vicious effect on stroke prognosis [41]. Hyperglycemia is found in up to 36.4% of patients with cerebral infarction, most of which are large arterial occlusions. Non-diabetic stroke patients with SIH have a worse prognosis than those with normal glucose levels and those with a history of diabetes [42]. It was reported that excessive glucose level is detrimental to brain structure and metabolism [33]. The pathogenesis of SIH in AIS is speculated to be the exacerbation of inflammation due to hyperglycemia, which in turn leads to increased blood–brain barrier permeability, edema, and hemorrhage. Under ischemic hypoxic conditions, hyperglycemia can cause anaerobic glycolysis and, consequently, acidosis. Furthermore, the accumulation of calcium and reactive oxygen species can result in neuronal damage as well as vasoconstriction caused by the low availability of nitric oxide. A prospective study [43] of 790 consecutively hospitalized AIS patients showed that those with SIH exhibited a higher incidence of adverse reactions and in-hospital mortality during hospitalization, and SIH can be used as a measure for evaluating the severity of AIS. Collectively, these findings indicate that early identification of SIH-related clinical manifestations or laboratory parameters may improve the prognosis of AIS patients.

### 4.3. Limitations

There are several limitations to this study. First, our analysis showed significant heterogeneity in SIH incidence among AIS populations, which may be attributed to the differences in population distribution and diagnostic criteria. Second, it was previously shown that persistent hyperglycemia is strongly associated with adverse outcomes in AIS patients. However, fasting glucose was used as the primary outcome measure, and continuous dynamic glucose monitoring was lacking for most of the SIH patients included in this study, which may impact the comprehensiveness of the results to a certain extent. Therefore, SIH inclusion criteria will need to be further refined and unified in future studies. Third, most of the study populations originated from Europe and Asia, and hence, the findings may not be applicable to other regions. Last, all of the studies included in this meta-analysis were observational studies, and further large-cohort longitudinal interventional studies are warranted.

## 5. Conclusions

Our systematic review showed that the incidence of SIH is relatively high in AIS, which indicates that SIH in AIS poses a serious public health problem. Therefore, greater measures should be taken for the prevention and control of SIH.

## Figures and Tables

**Table 1:** General characteristics of included studies. Refs [23,24,25,26,27,28,29,30,31,32,33,34,35].

No.	Author	Year	Country	Study Type	Sample Size	Number of Hyperglycemia	Gender (Male/Female)		Age		NOS Score
							Hyperglycemia	Non-Hyperglycemic	Hyperglycemia	Non-Hyperglycemic	
1	Berger	1986	Canada	Retrospective cohort	21	9	4/5	6/6	55	52.5	9
2	Toni	1992	Italy	Retrospective cohort	257	93	49/44	106/58	69.8	66	9
3	Szczudlik	2001	Poland	Retrospective cohort	197	95	49/46	55/47	71.6	67.9	9
4	Wang	2001	Australia	Retrospective cohort	333	57	/	/	/	/	9
5	Kyadav	2004	India	Retrospective cohort	16	3	/	/	/	/	9
6	Yong	2008	Finland	Retrospective cohort	487	79	39/40	247/161	69	66.5	9
7	Stead	2009	United States	Retrospective cohort	332	78	43/35	133/121	76.2	72.4	9
8	Dziedzic	2010	Poland	Retrospective cohort	224	32	16/16	90/102	75.5	69	9
9	Shimoyama	2014	Japan	Retrospective cohort	271	85	/	/	/	/	9
10	Mi	2017	China	Retrospective cohort	41	4	3/1	25/12	56.7	59.1	9
11	Tziomalos	2017	Greece	Retrospective cohort	537	68	27/41	193/276	81.5	79.5	9
12	Zonneveld	2017	Netherlands	Retrospective cohort	1676	338	186/152	770/568	72.5	72.5	9
13	Wu	2020	China	Retrospective cohort	160	36	11/25	46/78	37.1	35.9	9

## Data Availability

The datasets used and/or analyzed during the current study are available from the corresponding author upon reasonable request.

## Supplementary Materials

The following supporting information can be downloaded at: https://www.mdpi.com/article/10.3390/brainsci13040556/s1, PRISMA_2020_checklist, and Search History.

## Abbreviations

SIH, Stress-induced hyperglycemia; AIS, Acute ischemic stroke; NOS, Newcastle–Ottawa Scale; RR, Relative risk; WMD, Weighted mean difference; SMD, Standard mean difference.
