# Supplementary material for: Incidence of Stress-Induced Hyperglycemia in Acute Ischemic Stroke: A Systematic Review and Meta-Analysis
Source: Brain Sci. 2023 Mar 26;13(4):556. doi: 10.3390/brainsci13040556 (PMC10136900; doi:10.3390/brainsci13040556)
Supplement: Supplementary file 1 [file brainsci-13-00556-s001.zip › Search History.pdf]

PubMed Search History

Search number	Query	Sort By	Filters	Search Details	Results	Time
**7**	(("Ischemic Stroke"[Mesh]) OR ((((((((((Ischemic Stroke[Title/Abstract]) OR (Ischemic Strokes[Title/Abstract])) OR (Stroke, Ischemic[Title/Abstract])) OR (Ischaemic Stroke[Title/Abstract])) OR (Ischaemic Strokes[Title/Abstract])) OR (Stroke, Ischaemic[Title/Abstract])) OR (Acute Ischemic Stroke[Title/Abstract])) OR (Acute Ischemic Strokes[Title/Abstract])) OR (Ischemic Stroke, Acute[Title/Abstract])) OR (Stroke, Acute Ischemic[Title/Abstract]))) AND (("Hyperglycemia"[Mesh]) OR (((Hyperglycemia[Title/Abstract])) OR (Hyperglycemias[Title/Abstract])))			("Ischemic Stroke"[MeSH Terms] OR ("Ischemic Stroke"[Title/Abstract] OR "ischemic strokes"[Title/Abstract] OR "stroke ischemic"[Title/Abstract] OR "ischaemic stroke"[Title/Abstract] OR "ischaemic strokes"[Title/Abstract] OR "stroke ischaemic"[Title/Abstract] OR "acute ischemic stroke"[Title/Abstract] OR "acute ischemic strokes"[Title/Abstract] OR "ischemic stroke acute"[Title/Abstract] OR "stroke acute ischemic"[Title/Abstract])) AND ("Hyperglycemia"[MeSH Terms] OR ("Hyperglycemia"[Title/Abstract] OR "Hyperglycemias"[Title/Abstract]))	609	09:26:30
**6**	("Hyperglycemia"[Mesh]) OR (((Hyperglycemia[Title/Abstract])) OR (Hyperglycemias[Title/Abstract]))			"Hyperglycemia"[MeSH Terms] OR "Hyperglycemia"[Title/Abstract] OR "Hyperglycemias"[Title/Abstract]	73,832	09:25:57
**5**	("Ischemic Stroke"[Mesh]) OR ((((((((((Ischemic Stroke[Title/Abstract]) OR (Ischemic Strokes[Title/Abstract])) OR (Stroke, Ischemic[Title/Abstract])) OR (Ischaemic Stroke[Title/Abstract])) OR (Ischaemic Strokes[Title/Abstract])) OR (Stroke, Ischaemic[Title/Abstract])) OR (Acute Ischemic Stroke[Title/Abstract])) OR (Acute Ischemic Strokes[Title/Abstract])) OR (Ischemic Stroke, Acute[Title/Abstract])) OR (Stroke, Acute Ischemic[Title/Abstract]))			"Ischemic Stroke"[MeSH Terms] OR "Ischemic Stroke"[Title/Abstract] OR "ischemic strokes"[Title/Abstract] OR "stroke ischemic"[Title/Abstract] OR "ischaemic stroke"[Title/Abstract] OR "ischaemic strokes"[Title/Abstract] OR "stroke ischaemic"[Title/Abstract] OR "acute ischemic stroke"[Title/Abstract] OR "acute ischemic strokes"[Title/Abstract] OR "ischemic stroke acute"[Title/Abstract] OR "stroke acute ischemic"[Title/Abstract]	69,002	09:25:39
**4**	((Hyperglycemia[Title/Abstract])) OR (Hyperglycemias[Title/Abstract])			"Hyperglycemia"[Title/Abstract] OR "Hyperglycemias"[Title/Abstract]	50,567	09:24:33
**3**	"Hyperglycemia"[Mesh]	Most Recent		"Hyperglycemia"[MeSH Terms]	39,720	09:22:36
**2**	(((((((((Ischemic Stroke[Title/Abstract]) OR (Ischemic Strokes[Title/Abstract]))			"ischemic stroke"[Title/Abstract] OR "ischemic strokes"[Title/Abstract] OR	67,897	09:19:20

	OR (Stroke, Ischemic[Title/Abstract])) OR (Ischaemic Stroke[Title/Abstract])) OR (Ischaemic Strokes[Title/Abstract])) OR (Stroke, Ischaemic[Title/Abstract])) OR (Acute Ischemic Stroke[Title/Abstract])) OR (Acute Ischemic Strokes[Title/Abstract])) OR (Ischemic Stroke, Acute[Title/Abstract])) OR (Stroke, Acute Ischemic[Title/Abstract])			"stroke ischemic"[Title/Abstract] OR "ischaemic stroke"[Title/Abstract] OR "ischaemic strokes"[Title/Abstract] OR "stroke ischaemic"[Title/Abstract] OR "acute ischemic stroke"[Title/Abstract] OR "acute ischemic strokes"[Title/Abstract] OR "ischemic stroke acute"[Title/Abstract] OR "stroke acute ischemic"[Title/Abstract]		
1	"Ischemic Stroke"[Mesh]	Most Recent		"Ischemic Stroke"[MeSH Terms]	7,129	09:16:36

Embase Search History

No.	Query	Results	Date
#7	#3 AND #6	1270	20 Jul 2022
#6	#4 OR #5	132814	20 Jul 2022
#5	'hyperglycemia':ab,ti OR 'hyperglycemias':ab,ti	73253	20 Jul 2022
#4	'hyperglycemia'/exp	112199	20 Jul 2022
#3	#1 OR #2	115367	20 Jul 2022
#2	'ischemic stroke':ab,ti OR 'ischemic strokes':ab,ti OR 'stroke, ischemic':ab,ti OR 'ischaemic stroke':ab,ti OR 'ischaemic strokes':ab,ti OR 'stroke, ischaemic':ab,ti OR 'acute ischemic stroke':ab,ti OR 'acute ischemic strokes':ab,ti OR 'ischemic stroke, acute':ab,ti OR 'stroke, acute ischemic':ab,ti	112384	20 Jul 2022
#1	'ischemic stroke'/exp	15456	20 Jul 2022

Cochrane Library Search History

Search Name: CochraneLibrarySearchHistory
Date Run: 20/07/2022 16:00:02
Comment:

ID	Search	Hits
#1	MeSH descriptor: [Hyperglycemia] explode all trees	3243

#2 (Hyperglycemia):ti,ab,kw OR (Hyperglycemias):ti,ab,kw 8534

#3 MeSH descriptor: [Ischemic Stroke] explode all trees 365

#4 (Ischemic Stroke):ti,ab,kw OR (Ischemic Strokes):ti,ab,kw OR (Stroke, Ischemic):ti,ab,kw OR (Ischaemic Stroke):ti,ab,kw OR (Ischaemic Strokes):ti,ab,kw 16947

#5 (Stroke, Ischaemic):ti,ab,kw OR (Acute Ischemic Stroke):ti,ab,kw OR (Acute Ischemic Strokes):ti,ab,kw OR (Ischemic Stroke, Acute):ti,ab,kw OR (Stroke, Acute Ischemic):ti,ab,kw 16847

#6 #1 OR #2 9662

#7 #3 OR #4 OR #5 16980

#8 #6 AND #7 135

Web of Science Search History

Session Queries

Build a new query based on your searches in this session.

0/3 Combine Sets Clear History

3	#2 AND #1	2,236	Add to query
2	(TS=(Hyperglycemia)) OR TS=(Hyperglycemias)	121,307	Add to query
1	((((((((TS=(Ischemic Stroke)) OR TS=(Ischemic Strokes)) OR TS=(Stroke, Ischemic)) OR TS=(Ischaemic Stroke)) OR TS=(Ischaemic Strokes)) OR TS=(Stroke, Ischaemic)) OR TS=(Acute Ischemic Stroke)) OR TS=(Acute Ischemic Strokes)) OR TS=(Ischemic Stroke, Acute)) OR TS=(Stroke, Acute Ischemic)	181,433	Add to query
